# The Homœopathic Club of Denver, Col.

**Published:** 1894-04

**Authors:** 


### THE HOMŒOPATHIC CLUB OF DENVER, COL.

The annual meeting of this Club was held on Monday evening, January 22d, at their rooms in the Brown Palace Hotel, President Kinley in the chair. There was an unusually large attendance.

The members of the Club have done a large amount of charitable work during the time since the Club was organized, and the reports read for last year show that they are doing a large amount of work of this kind.

Dr. W. A. Burr reported for the Deaconess' Home and Hospital Committee.

Dr. J. P. Willard made the report for the Haymarket Mission Dispensary.

Dr. J. Wylie Anderson reported that the inmates of the Denver Orphans' Home were all in good health, and that there had been no deaths among the inmates during the year. He also stated that since the Homœopathic School took charge there have been no cases of ringworm among the children, although before that time this had been a great bane to the directors.

The Ladies' Relief Home report was presented by Dr. Shannon.

The report from the W. C. T. U. Day and Night Nursery was presented by Dr. Alexander.

The Secretary's report showed that twenty-five meetings had been held during the year, twenty-three regular and two special ones. There were nineteen papers read before the members at the various meetings, and the papers were both interesting and instructive and the discussions were general.

President Kinley then presented his address, consisting of a *résumé* of the work done, and giving some very practical suggestions in the way of new work to be undertaken by the members during 1894.

The election was then proceeded with, and the following officers were chosen: President, Dr. J. Wylie Anderson; Vice-President, Dr. C. W. Enos; Secretary, Dr. S. F. Shannon; Treasurer, Dr. S. S. Smythe, Censors, Drs. S. S. Kehr, J. B. Kinley, E. H. King; Delegate to the American Institute of Homœopathy, Dr. W. A. Burr.

